# Exploring the use of Rasch modelling in “common content” items for multi-site and multi-year assessment

**DOI:** 10.1007/s10459-024-10354-y

**Published:** 2024-07-08

**Authors:** David Hope, David Kluth, Matthew Homer, Avril Dewar, Rikki Goddard-Fuller, Alan Jaap, Helen Cameron

**Affiliations:** 1 Medical Education Unit, The Chancellor’s Building, College of Medicine and Veterinary Medicine, The University of Edinburgh, 49 Little France Crescent, Edinburgh, Scotland, EH16 4SB UK; 2 Leeds Institute of Medical Education, Leeds School of Medicine, Worsley Building, University of Leeds, Woodhouse, Leeds, LS2 9JT UK; 3 Christie Education, The Christie NHS Foundation Trust, Manchester, M20 4BX UK; 4 Aston Medical School, Aston University, 295 Aston Express Way, Birmingham, B4 7ET UK

**Keywords:** Rasch measurement, Assessment, Psychometrics, Medical licensing examination, Validity

## Abstract

Rasch modelling is a powerful tool for evaluating item performance, measuring drift in difficulty over time, and comparing students who sat assessments at different times or at different sites. Here, we use data from thirty UK medical schools to describe the benefits of Rasch modelling in quality assurance and the barriers to using it. Sixty “common content” multiple choice items were offered to all UK medical schools in 2016-17, and a further sixty in 2017-18, with five available in both years. Thirty medical schools participated, for sixty total datasets across two sessions, and 14,342 individual sittings. Schools selected items to embed in written assessment near the end of their programmes. We applied Rasch modelling to evaluate unidimensionality, model fit statistics and item quality, horizontal equating to compare performance across schools, and vertical equating to compare item performance across time. Of the sixty sittings, three provided non-unidimensional data, and eight violated goodness of fit measures. Item-level statistics identified potential improvements in item construction and provided quality assurance. Horizontal equating demonstrated large differences in scores across schools, while vertical equating showed item characteristics were stable across sessions. Rasch modelling provides significant advantages in model- and item- level reporting compared to classical approaches. However, the complexity of the analysis and the smaller number of educators familiar with Rasch must be addressed locally for a programme to benefit. Furthermore, due to the comparative novelty of Rasch modelling, there is greater ambiguity on how to proceed when a Rasch model identifies misfitting or problematic data.

**Supplementary Information:**

The online version contains supplementary material available at 10.1007/s10459-024-10354-y.

## Introduction

A key goal of assessment in medical education is to ensure that doctors will be fit to practise medicine (Cox et al., 2007; Norcini, 1999). Due to this, post-assessment evaluation is needed to determine the utility and defensibility of assessment– especially in high-stakes situations where assessment is a prerequisite for registration as a doctor (Boursicot et al., 2006). Such post-hoc evaluations can inform medical educators of the assessment’s reliability and validity, the presence of poorly performing items, flaws in candidate knowledge, opportunities to minimise costs, the possibility of assessor bias, gaps in blueprints and the presence of testwise behaviour– among a range of other features (Chen et al., 2020; Pell et al., 2010, 2013; Tavakol & Dennick, 2012). Improving the quality of post-assessment evaluation can therefore improve the quality of assessment itself.

A particular challenge for those working in assessment is the need for authentic assessment tools aligned to the evolving landscape of clinical practice and relevant tools/skills to quality assure this assessment. Three issues of growing relevance in assessment are particularly significant in driving the need for innovation in post-assessment evaluation.

Firstly, there is an increasing trend towards repeated exposure to the same content (Wrigley et al., 2012), or cross-institutional assessment in the form of a shared “national licensing assessment” which candidates have to pass before graduating (Allawi et al., 2016; Cuddy et al., 2017; Schuwirth et al., 2010). Comparing results across cohorts and sites is challenging, but essential to properly evaluate both the performance of content and the candidates in these types of assessments.

Secondly, standard setting methods have often focused on hypothetical borderline candidates (Ricker, 2006), but post-evaluation assessment typically reports the average candidate performance per item, and does not estimate how a truly borderline candidate would perform on each item. This complicates item review, makes it harder to select items that are particularly discriminating for borderline candidates, and leads to difficulties in evaluating gaps between predicted and actual performance; or even whether the standard setting process is consistent over time (Homer & Darling, 2016).

Finally, all assessment is influenced by context. The size of assessment, the size of a cohort, and item location in the test, can exert a considerable impact on the utility of item statistics and the quality of information derived from them. However, although there are increasing calls for psychometric models to better align with the needs of medical education assessment (Schuwirth & van der Vleuten, 2006), relatively little is known about whether complex analytical processes are feasible when applied to undergraduate medical education, where sample sizes and item numbers may be small and the range of candidate abilities may be restricted (Homer, 2021; Hope et al., 2021). Institutions may therefore vary in their ability to use such novel tools and be in the difficult position of considering new statistical techniques that have not been tested in their context.

Historically, the quantitative aspects of post-evaluation assessment have been delivered via Classical Test Theory (CTT). The advantages and disadvantages of this method has been well-described (De Champlain, 2010) but in brief CTT describes the reliability of assessment (usually in the form of Cronbach’s alpha) and provides item information on facility (candidate performance) and discrimination (whether the item can identify those who performed well or poorly overall). CTT has several key problems: it is heavily dependent on the test structure, with discrimination and facility values varying based on sample size and test length, making comparisons between test forms or cohorts very difficult (Tavakol & Dennick, 2013). Values are usually reported in aggregate form as an average, so it is difficult to identify the particular needs of borderline candidates, or to compare different cohorts (to test for e.g. improvements over time, or collusion) except using crude mean-score comparisons. This in turn makes it challenging to reflect on gaps between standards set and performance achieved or to meaningfully monitor item change over time (De Champlain, 2010; Tavakol & Dennick, 2012).

By contrast, Item Response Theory (IRT) models such as Rasch offer significant advantages in a way that may improve post-assessment evaluation. Rasch models have been described in detail elsewhere (Homer & Darling, 2016; Rasch, 1960; Tavakol & Dennick, 2012) but in brief, Rasch modelling first assesses the dimensionality of the test– can it be described as having a single, underlying domain of ability or are there several? - then proceeds to measure student ability and item difficulty on the same scale, and assume the same discrimination parameter. Using this scale (logits) whereby higher logits indicate higher ability and lower logits indicate lower abilities, it is possible to estimate the performance of any candidate for any item (Tavakol & Dennick, 2013). While more complex than CTT, Rasch is also simpler than 2- and 3- parameter IRT models that seek to model additional sources of variance (including candidate guessing) and have stricter sample size requirements. The specific requirements and advantages of each form of IRT have been described elsewhere (Andrich, 2004; Bock, 1997).

Rasch computes item difficulties in a form that is independent of the sample or assessment used, giving greater comparability across assessments and across time. Importantly, Rasch can use “anchor items” to compare items that have never been used in the same assessment. If item A is used in test 1, and item B is used in test 2, but item C is used in both tests, item C can be used as an “anchor” to compare Items A and B– and to compare the candidates sitting test 1 and test 2 using the same universal scale (McManus et al., 2014). Applied effectively, anchor items can be used to compare many cohorts at many sites across many non-shared items– allowing medical educators to compare candidates in a range of environments.

Besides this, the ability to estimate performance across the ability scale allows Rasch to estimate performance for borderline candidates, allowing for a much more direct examination of mismatches between the set standard and actual performance. Rasch also provides a range of detailed fit statistics that can be used to identify redundant items (that is, items which can be removed with no loss to the assessment), report on the breadth of ability being tested (thereby highlighting tests that drift into being too difficult, or too far from the passing standard) and identify whether an item is an effective discriminator for candidates close to average ability or at the extremes (Loyd & Hoover, 1980; Van der Linden, 2016). By extending the model to examine multiple groups, it is possible to examine whether the overall assessment– and each individual item– is fair to studied groups, which greatly increases opportunities to monitor fairness in assessment (Hope et al., 2018). In summary, Rasch provides a superior, longitudinal overview of item quality assurance and greater insights into multi-site and multi-year assessment with potential benefits for repeated assessments (Wrigley et al., 2012).

Despite these considerable advantages, almost all researchers acknowledge Rasch requires a greater understanding of complex statistics and is familiar to a much smaller range of medical educators than CTT methods (Tavakol & Dennick, 2012). Furthermore, the assumptions involved in psychometric approaches often favour larger sample sizes, with larger item pools in each assessment, which may not be feasible in reality (Homer & Darling, 2016). Institutions may vary in their ability to utilise advanced methods such as Rasch depending on test length or cohort size, which they have only limited control over.

In the present study, we developed a Rasch analysis and associated reporting method designed to support medical educators who have no familiarity with Rasch. We utilised Rasch as opposed to more complex IRT models for two reasons. Firstly, Rasch is particularly beneficial in that it allows for the creation of a single scale that works the same way for all candidates and can be used to determine what those candidates do and do not know across ability levels and sittings (Stemler & Naples, 2021) and so was more useful given our goal of informing standards. Secondly, the comparative simplicity of Rasch compared to other IRT models made it a useful introduction to non-CTT methods for novices.

We carried out the Rasch analysis on “common content” MCQs developed by the Medical Schools Council Assessment Alliance (MSCAA) to compare candidates at 30 UK medical schools over two years, to evaluate the utility of Rasch information in enhancing standard setting, evaluating anchor items, equating, and monitoring changes in performance over time.

## Methods

### Context and study design

In the United Kingdom, medical schools develop their own teaching and assessment, but are regulated by the General Medical Council (GMC) in relation to standards and high-level outcomes (General Medical Council, 2018). Typically, medical students spend five or six years studying an undergraduate degree programme, and, by the end of their programme, sit written and clinical examinations designed to ensure readiness for their role as a new doctor. While medical schools differ in the quantity of assessment (McManus et al., 2008) all medical schools described here delivered both written and clinical components for final assessment.

Our study uses “single best answer” multiple choice questions developed by the UK Medical Schools Council Assessment Alliance (MSCAA) as “common content” that can be used by all schools in their graduating knowledge test assessments. 60 core items were available in 2016-17, and 60 in 2017-18, with five individual items used in both sessions. Items were curated by the MSCAA Final Clinical Review Group, to which all UK medical schools contributed, and were blueprinted against GMC Outcomes for Graduates and content areas. 23 individual content areas were used, with 2.6 items on average per area. Schools could choose to use all, some, or none of the items, and all items were delivered to students as part of the final written assessment at their medical school (Taylor et al., 2017; Yeates et al., 2019). Further information describing the common content project can be found elsewhere (Hope et al., 2021; Taylor et al., 2017).

### Participants

The common content project was open to all UK medical schools. In 2016-17, 30 medical schools used a mean of 52.6 common content items, which were delivered to 7,177 medical students. In 2017-18, 30 medical schools used a mean of 52.8 common content items, which were delivered to 7,165 medical students, for a total of 14,342 sittings within the present study.

### Ethics

The University of Edinburgh Medicine and Veterinary Medicine ethics committee approved this study. All details were anonymous, and the researchers could not identify individual medical students, or individual medical schools, at any point.

### Data analysis

We initially tested the number of dimensions for each school using parallel analysis (Crawford et al., 2010) and then tested goodness of fit via the Andersen L-R test and the Wald test (Glas & Verhelst, 1995). Traditionally, a dataset would be expected to exhibit unidimensionality and not violate goodness of fit measures to be suitable for this form of Rasch modelling, but we ran the Rasch model even in cases where the assumptions were not met to see whether the resultant data could still be useful to schools, thereby taking a liberal approach to the thresholds for model adequacy.

Parallel analysis is a method of identifying the number of dimensions (or factors) in a dataset (Crawford et al., 2010). Where multiple dimensions are found, schools were informed so as to be able to identify if some clusters of knowledge were being over- or under- represented in their context and whether their assessments could be reliably summed up as a single score. The goodness of fit statistics fundamentally check whether the quality of measurement is high enough (Glas & Verhelst, 1995) and as such cases where assumptions were violated encouraged revisions to content and teaching.

We analysed each school’s dataset separately rather than pooling all data into a single analysis. There were several reasons for this. On a practical level, this allowed us to give granular feedback to each school and to identify school-level differences in either fit statistics or performance. Given the schools were unequal in size, some very large schools might have disproportionately influenced overall values if pooled. Secondly, pooling data would have required some items to have either significant amounts of missing data, or for those values to be estimated, which would have made them less useful to standard setters at those schools.

Following testing for assumptions, we calculated the item difficulty and associated item fit statistics to explore item performance. Besides simple measures of item difficulty, we also calculated infit and outfit statistics, which help estimate how useful the item is at discriminating between candidates near the mean score (infit) and at the extremes of the distribution (outfit). Additionally, infit and outfit can measure not just whether the item is misfitting and adding error to the model, but also whether the item adds so little value to the model that it can be removed without further issue. Given the focus in this paper is on evaluating the overall applicability of Rasch modelling to these types of datasets, we do not discuss item statistics in detail.

Next, we plotted Item Characteristic Curves (ICCs) for all items. ICCs are a useful tool for examiners seeking to visualise the association between ability, and the likelihood of a candidate answering an item correctly. The results take the form of an s-shaped curve, and both the location of the slope and its shape can be informative as to the item’s difficulty and where on the ability curve it best discriminates between candidates.

As an addendum to this, we generated an item map which provides confidence intervals for estimates of difficulty. This is important, as it helps assessors understand the uncertainty around model fit values, and to be aware of the level of uncertainty associated with them.

Finally, we conducted a horizontal and vertical chain equating model (Sansivieri et al., 2017). By using anchor items shared across schools, we were able to estimate how a school would have performed on items not sat by any candidate in that school. This analysis estimated the relative performance of candidates across schools on a universal scale, using an arbitrarily selected school as the initiating anchor. As part of our vertical chain equating, we were able to model item drift– looking at the difference between item performance across years, within and between institutions. We were able to measure not just changes in average scores, but also whether the item curves changed significantly over time. This allowed us to note whether, for example, some schools were effective at raising the level of their borderline candidates for some content or whether performance appeared stable.

Throughout, the main emphasis of our approach was on evaluating whether the type of data available here met the criteria for Rasch modelling, and to identify whether the additional work required to undertake Rasch modelling was sufficiently useful in supporting post-hoc assessment evaluation. However, we highlight here how the information provided was used by schools.

As such, detailed values such as fit statistics or equating tables are referenced only when necessary to address that key issue. Throughout the results, we highlight illustrative examples of useful elements of Rasch modelling, without providing an exhaustive record of all the output generated. A list of R packages used in the analysis can be found in Electronic Supplement 1.

## Results

Schools generally met the requirements for unidimensionality and goodness of fit measures for the overall model. Of the sixty sittings, only three failed to exhibit unidimensionality and eight violated the goodness of fit measures after controlling for multiple comparisons. Generally, schools that violated these assumptions had either very small sample (cohort) sizes, or uniformly high performance (which range-restricted the data). This information was fed back to schools with issues so they could compare-and-contrast their results with those of other schools and make changes for future years.

As previously noted, these schools continued to be included in analyses described here and the item fit statistics and confidence intervals did not noticeably differ from those that met the criteria.

In 2016-17, mean item difficulty ranged from − 2.08 to 2.47 with a mean SD of 0.55. On average, 26.38 schools sat each item. In 2017-18, mean item difficulty ranged from − 2.45 to 1.84 with a mean SD of 0.55 and, on average, 26.28 schools sat each item.

**Fig. 1:**
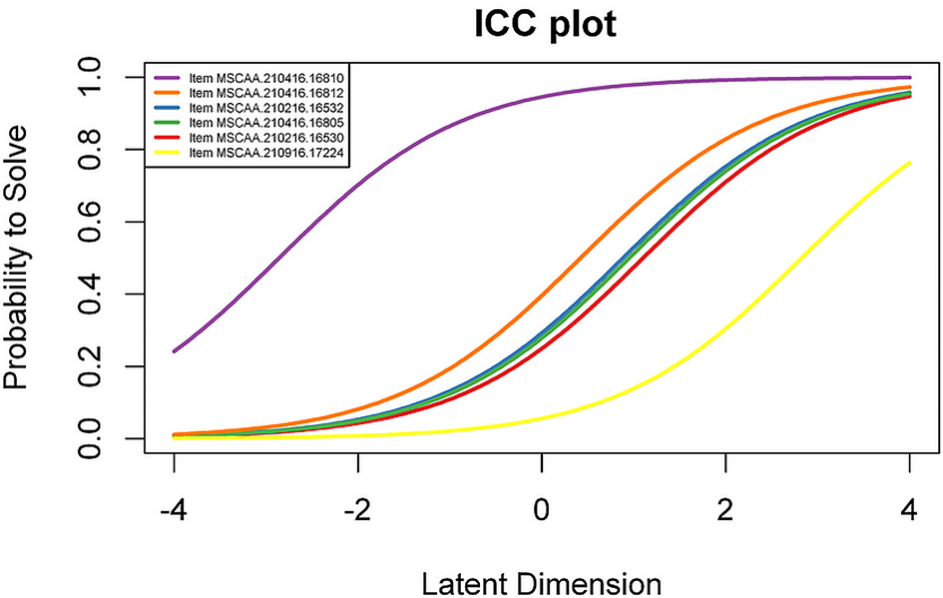
Item Characteristic Curve (ICC) plot. **Note** In the ICC plot, “latent dimension” represents ability, with higher ability corresponding to more difficult items. “Probability to solve” indicates the likelihood of the candidate answering the question correctly

Items varied in difficulty, and exhibited acceptable fit statistics: values of between 0.5 and 1.5 for infit and outfit (Tavakol & Dennick, 2013). Subsequently, we generated ICC plots of all items, for each school, to help assessors compare the standard they expected of a borderline candidate to the actual performance of a borderline candidate. (see Fig. 1).

In this plot, purple represents an extremely easy item, while yellow represents a very difficult item, with the rest somewhere in between. These plots– along with variants highlighting the popularity of distractor items at different levels of ability– were given to schools to help evaluate the estimated vs. actual difficulty and to help revise items where necessary. In Fig. 1., for example, schools could then evaluate whether the purple item was too easy, or the yellow item too difficult, whether the distractors were functioning adequately, and to focus such investigations on borderline candidates.

An inspection of the confidence intervals in the item map showed no problems requiring the removal of items but highlighted that uncertainty around the true score could be relatively higher in datasets with small sample sizes and few items. This also meant that some items were in a statistical sense redundant– they provided no additional information on candidate ability and could be removed.

**Fig. 2:**
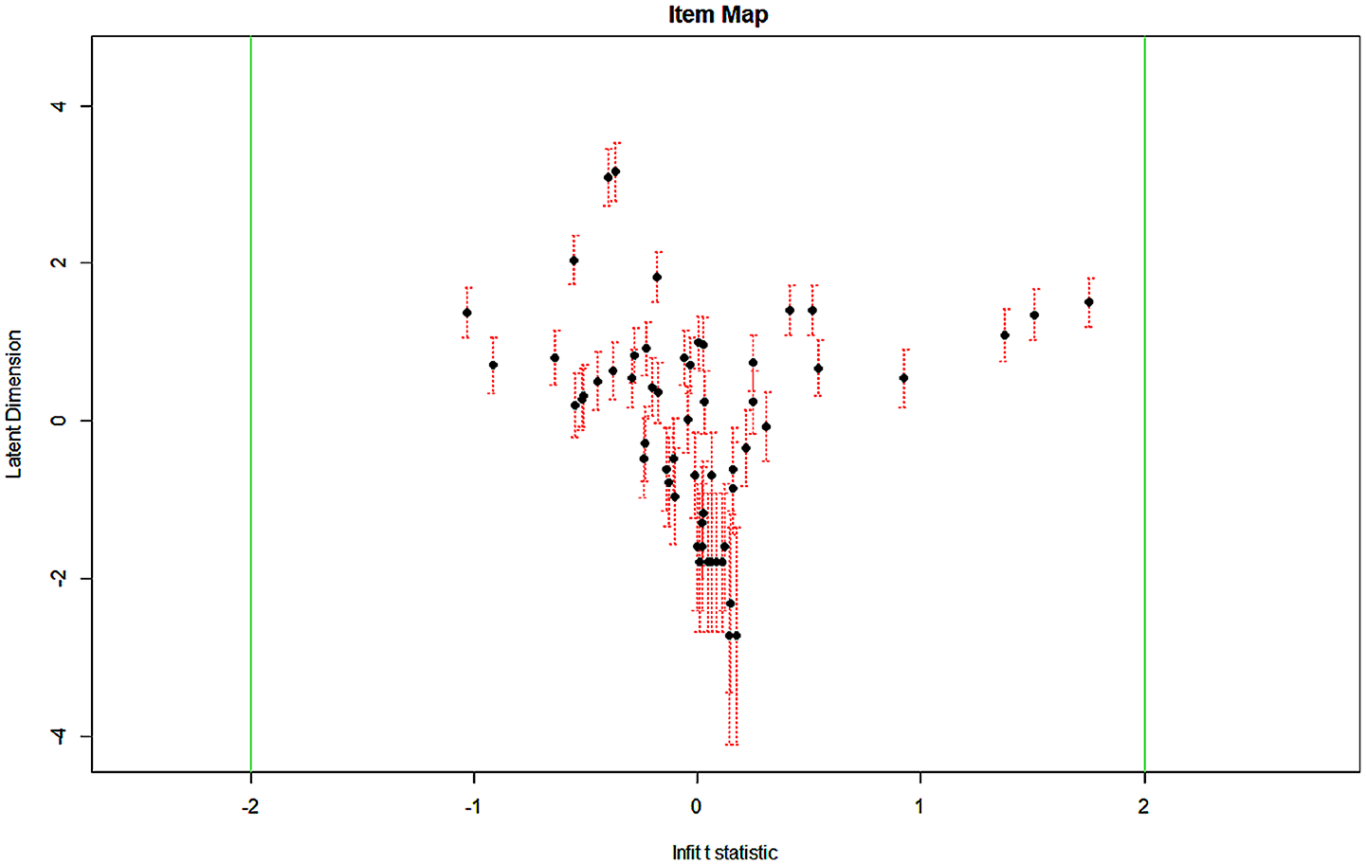
Item Map. **Note** Each data point represents an item from the 2016-17 dataset. Latent dimension refers to item difficulty, while infinit t statistic describes how much information about candidates is added by the item

Any items outside the green line (-2 or + 2) would be adding minimal information to the assessment and would be considered for removal. Higher scores on the latent dimension indicate items testing higher levels of ability. This information was passed to schools so they could determine whether their selection of items matched the level of difficulty they intended. Note in Fig. 2 many items clustered around the − 2 level of the latent dimension, indicating the content targeted the borderline or marginally failing candidate.

The horizontal chain equating model, that allowed us to compare performance across schools even when they did not sit identical assessment, can be found in Electronic Supplement 2. In general, for each year, school performance varied widely. For example, if the anchor group received a score of 34 (just over 60%), other schools would be expected to score between a low of 7.75 and a high of 36.21 on the same assessment– implying very high variability across schools.

A comparison of items across schools demonstrated that schools differed not just in the overall performance on the item, but the relative performance of candidates at different levels of ability.

**Fig. 3:**
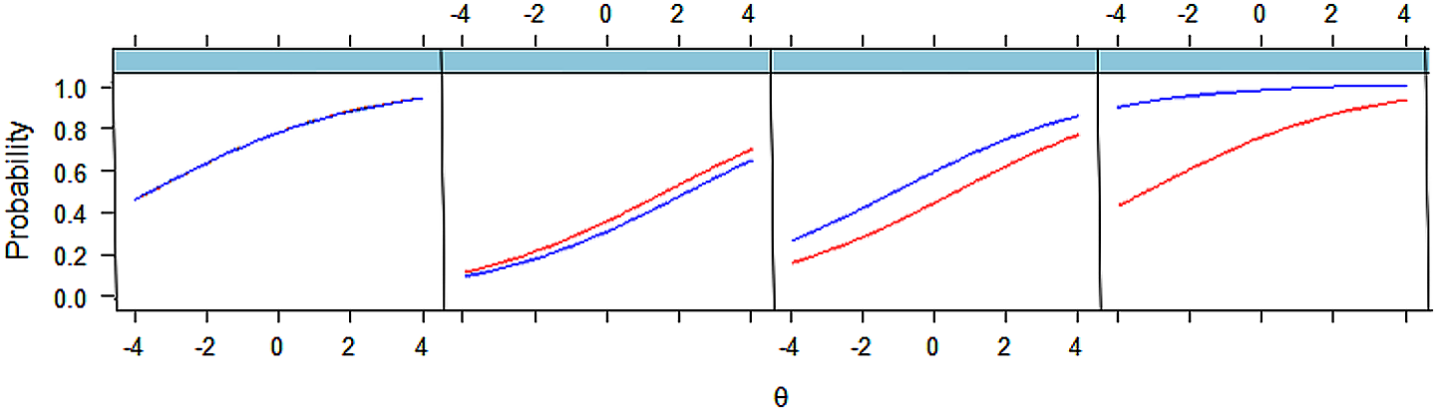
Horizontal equating

Figure 3: Four comparisons of items sat by two different schools, in the same session. From left to right, these show no differences, very minor differences, a difference in mean performance but not the curve, and a difference in mean performance and the curve.

**Fig. 4:**
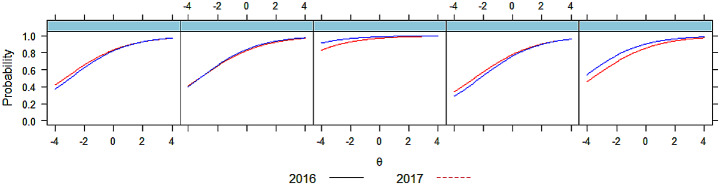
Vertical equating model

Our vertical equating model allowed us to compare the same items at the same schools across two sittings. This provided information on whether drift occurred between sittings. On inspection, no items drifted sufficiently to result in meaningful changes to pass rates, indicating items had acceptable stability. Figure 4 illustrates this trend.

Figure 4: Five comparisons of items, sat by the same school in both sessions.

The equating plots were given to schools so they could identify their candidate scores in relation to other cohorts at an item level, and to identify content areas where they were improving (or not). The stability of item fit statistics (see e.g. the first panel of Fig. 4) provided reassurance to schools that items could be predictably selected by difficulty in advance.

## Discussion

Rasch modelling is a powerful tool for evaluating the performance of items, testing the accuracy of standards, and monitoring differences between schools and across time. Many of the issues discussed here cannot easily be addressed by Classical Test Theory, and so adopting Rasch (or other IRT methods) is advantageous, assuming the capacity to run the analyses and interpret them is available. Doing so is particularly valuable when it is necessary to make multisite or multiyear comparisons in, for example, progress tests, multi-institutional or national-level assessments.

Our findings generally align with those of past research, particularly those discussing the potential efficacy of Rasch modelling (Homer & Darling, 2016; Rasch, 1960; Tavakol & Dennick, 2013). The use of equating to compare cohorts is well-established in the postgraduate assessment environment (McManus et al., 2014) and– if data are sufficiently robust– extending it to undergraduate medical education has a number of advantages. In light of the growing tendency for medical schools to be compared in a very detailed, granular way (Norcini et al., 2014), having the capacity to routinely evaluate against other schools and cohorts will be very helpful.

Other aspects of our findings were more mixed. As noted, some of the data did not meet the traditional cutoffs for Rasch modelling in terms of unidimensionality or goodness of fit. There has historically been an issue in medical education where educational data does not necessarily meet such criteria, but are analysed anyway e.g. the tendency for OSCEs with historically low reliability measures to still be considered acceptable for high-stakes decision making (Brannick et al., 2011). A key problem for the future application of IRT-methods is in determining what thresholds are acceptable within medical education, and whether decisions based on these analyses will be considered defensible even if (as in the case of many CTT methods) they do not fully meet traditional statistical assumptions.

This paper represents a significant contribution to our understanding of how Rasch can be applied in a real-world example, using thirty schools, over ten thousand sittings, and two years of data. All items used were reviewed by experts in the relevant domain and approved by individual schools. Schools generally– but not universally– produced data suitable for Rasch analysis and equating. As such, it suggests that real-world data will be suitable for these analyses in the vast majority of cases. Importantly, while statistically complex, it shows it is feasible for schools to routinely compare their performance against other schools, and evaluate item drift over time, while undertaking a more robust system of psychometric item evaluation and standard setting review compared to CTT methods. As highlighted in the introduction, this brings multiple insights (and benefits) to a quality improvement approach to assessment.

However, the study also had some limitations. Schools chose which items to embed in their final assessment, and the non-shared content could not be examined here. The causes of variability in performance across schools, or why some schools failed to produce unidimensional datasets that met goodness of fit criteria, cannot be identified from this study.

There are several notable possibilities for future work. The first is to expand equating across more years of data, to learn more about school-level differences, and item drift over time. This could increase our understanding of how stable item difficulty is, and how frequently items need to be revised. Secondly, given the complexity of Rasch modelling, it would be beneficial to explore how to better support assessors engaging with complex psychometric data. Finally, we did not formally test for Differential Item Functioning (DIF), as the work was intended to be evaluative, but doing so in future would provide further insights into the stability of anchor items. (Hope et al., 2018; Wang & Yeh, 2003)

In summary, Rasch modelling presents a number of opportunities for assessors working in medical education. However, the resources required to routinely implement it, and the relatively high bar of the technical aspects will make widespread usage challenging.

All authors have approved the final version.

## Electronic supplementary material

Below is the link to the electronic supplementary material.

Supplementary Material 1

Supplementary Material 2

## Data Availability

No datasets were generated or analysed during the current study.
