# Supplementary material for: Exploring the use of Rasch modelling in “common content” items for multi-site and multi-year assessment
Source: Adv Health Sci Educ Theory Pract. 2024 Jul 8;30(2):427–38. doi: 10.1007/s10459-024-10354-y (PMC11965148; doi:10.1007/s10459-024-10354-y)
Supplement: Supplementary file 2 — Supplementary Material 2 [file 10459_2024_10354_MOESM2_ESM.docx]

R packages used in the analysis :

dplyr (https://dplyr.tidyverse.org/)

eRm (https://cran.r-project.org/web/packages/eRm/eRm.pdf)

flextable (https://cran.r-project.org/web/packages/flextable/flextable.pdf)

ltm (https://cran.r-project.org/web/packages/ltm/ltm.pdf)

plink (https://cran.r-project.org/web/packages/plink/plink.pdf)

plyr (https://cran.r-project.org/web/packages/plyr/plyr.pdf)

psychometric (https://cran.r-project.org/web/packages/psychometric/psychometric.pdf)
